# 

**Published:** 1873-06

**Authors:** 


***To Correspondents.***

Some of our correspondents may miss replies, for the somewhat personal reason that a sneak thief invading our sanctum, took off with him not only a portion of our wearing apparel, memorandum book, etc., but also a goodly packet of letters, some unanswered. Replies forwarded on notice.

***Homœopathy in the Michigan University.***

The Regents decree that hereafter all diplomas shall be signed only by the President and Secretary of the University. An adroit dodge.

